# Supplementary material for: A Novel Hydrothermal CdS with Enhanced Photocatalytic Activity and Photostability for Visible Light Hydrogenation of Azo Bond: Synthesis and Characterization
Source: Nanomaterials (Basel). 2023 Jan 19;13(3):413. doi: 10.3390/nano13030413 (PMC9921911; doi:10.3390/nano13030413)
Supplement: Supplementary file 1 [file nanomaterials-13-00413-s001.zip › nanomaterials-2149774-supplementary.pdf]

SUPPLEMENTARY INFORMATION

A novel hydrothermal CdS with enhanced photocatalytic activity and photostability for visible light hydrogenation of azo bond: synthesis and characterization

Martina Milani [1], Michele Mazzanti [1], Giuliana Magnacca [2], Stefano Caramori [1], Alessandra Molinari [1*]

[1] Dipartimento di Scienze Chimiche, Farmaceutiche ed Agrarie, Università di Ferrara, Via Luigi Borsari 46, 44121, Ferrara, Italy
[2] Dipartimento di Chimica, Università di Torino, via P. Giuria 7, 10125 Torino, Italy
* alessandra.molinari@unife.it

Figure S1. N_2 adsorption/desorption isotherms of CdS-HT (circles), CdS-HTa400 (triangles) and commercial CdS (squares)

Figure S2. XPS survey spectra of CdS-HT, CdS-HTa400 and commercial CdS

Table S1. Atomic composition (% at.) obtained from XPS of CdS-HT and commercial CdS

Figure S3. Adsorption spectrum of commercial CdS

Figure S4. Tauc Plots and band gap evaluation of CdS-HT (a), CdS-HTa400 (b) and commercial CdS (c)

Figure S5. UV-vis spectra of MO dissolved in H_2O/EtOH (10%) solution during irradiation ($\lambda >$ 400 nm) of CdS-HTa400 under deaerated conditions.

Figure S6. XRD pattern of commercial CdS annealed at 400°C. For comparison XRD pattern of commercial XRD (already shown in Figure 1) is reported here for an easier comparison.

Figure S7. MO conversions (%) obtained by irradiating ($\lambda >$ 400 nm) deaerated suspensions of CdS-HTa400 or commercial CdS (10 or 15 min respectively) in four consecutive photocatalytic runs.

Figure S8. (a) Survey spectra, (b) Auger signal, (c) Cd 3d signal and (d) S 2p signal of CdS-HTa400as prepared and after use

Table S2. Atomic composition (% at.) obtained from XPS of CdS-HTa400 before and after use.

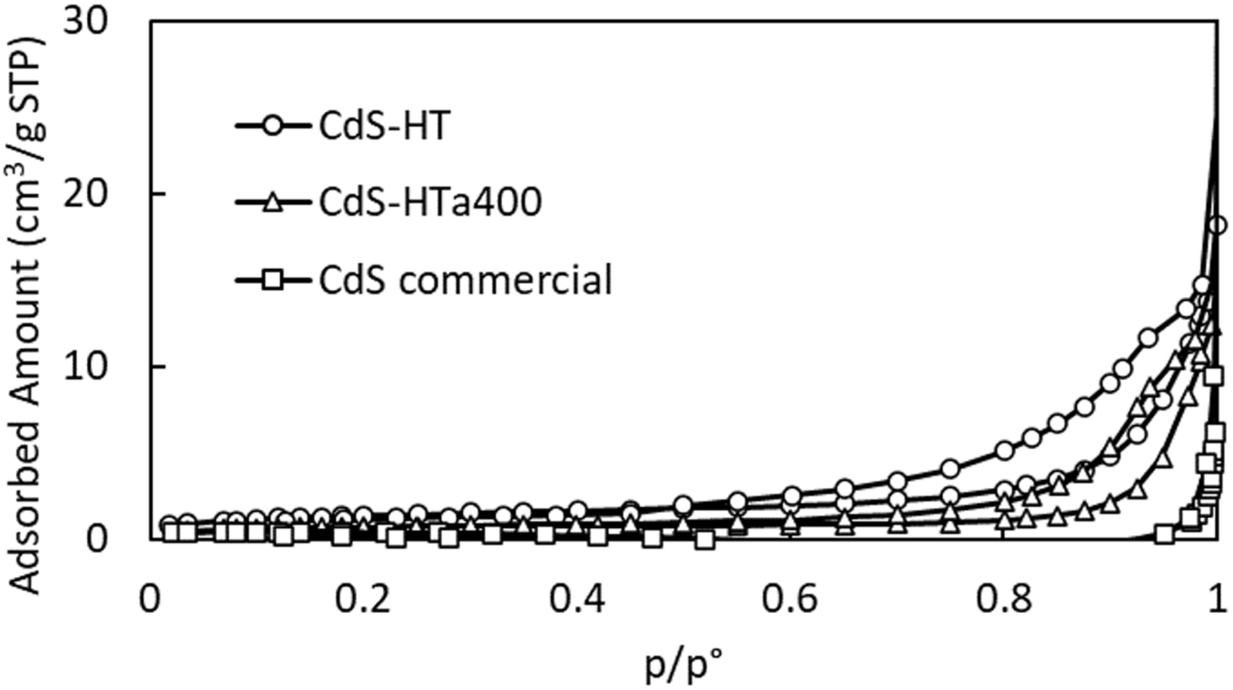

Figure S1.

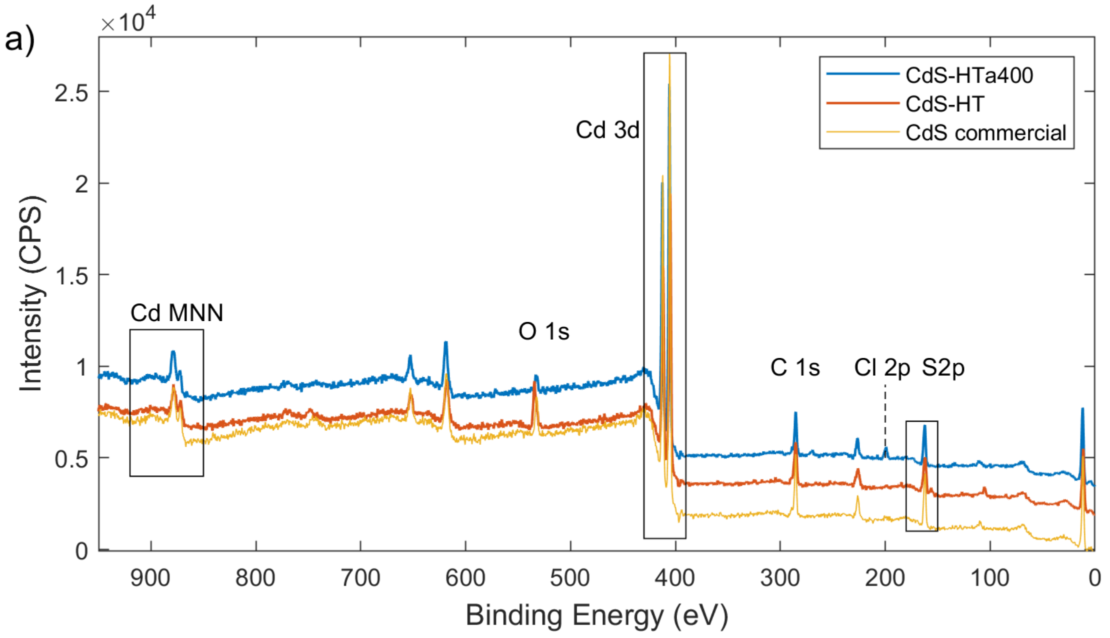

Figure S2.

Table S1

Elements	C 1s	O 1s	Cd 3d 5/2	S 2p	Cl 2p	Cd/S
CdS commercial	45.1 ± 1.0	12.7 ± 0.7	22.5 ± 0.8	19.7 ± 0.8	-	1.1 ± 0.1
CdS-HT	44.0 ± 1.0	18.6 ± 0.7	18.3 ± 0.8	19.1 ± 0.8	-	1.0 ± 0.1

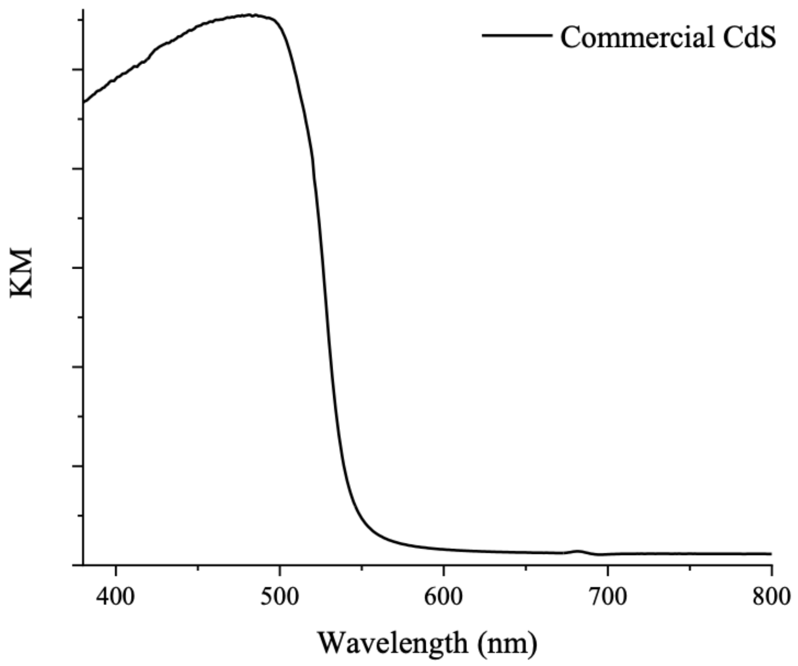

Figure S3.

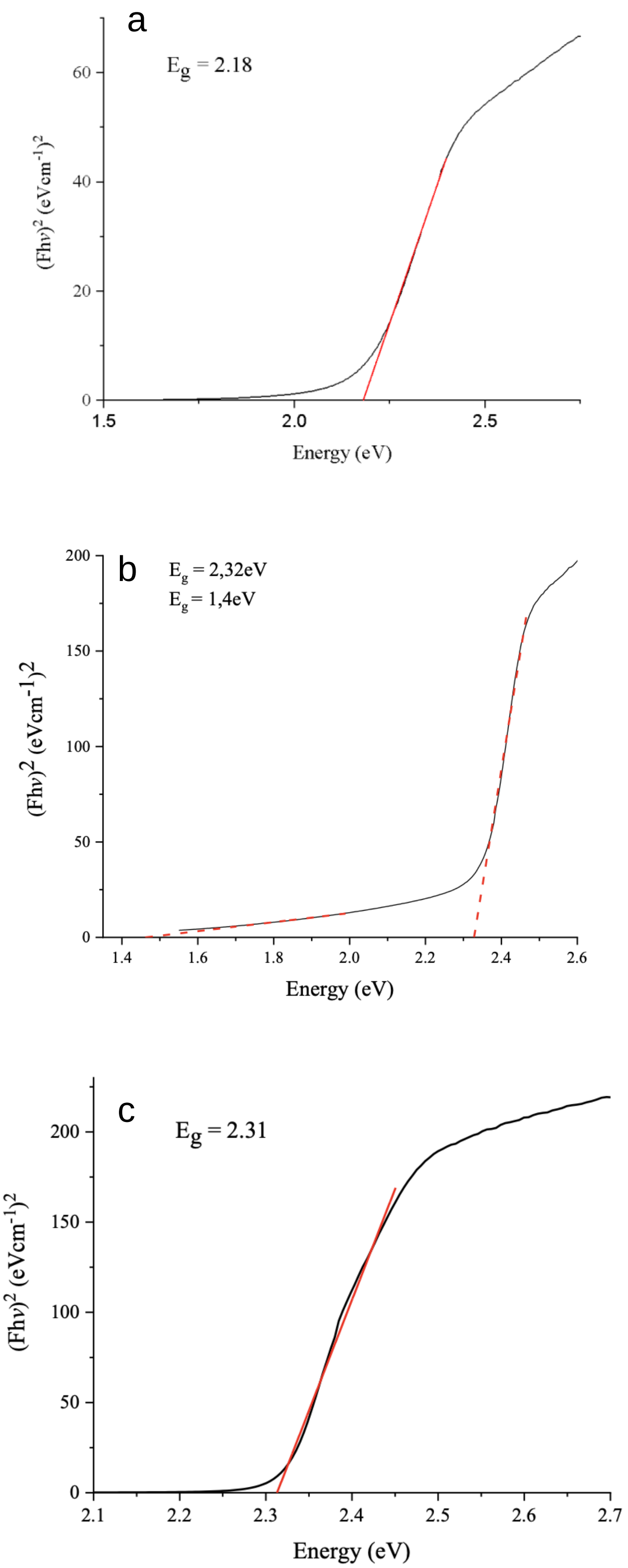
a
Eg = 2.18
(Fhν)2 (eVcm-1)2
Energy (eV)
b
Eg = 2,32eV
Eg = 1,4eV
(Fhν)2 (eVcm-1)2
Energy (eV)
c
Eg = 2.31
(Fhν)2 (eVcm-1)2
Energy (eV)

Figure S4.

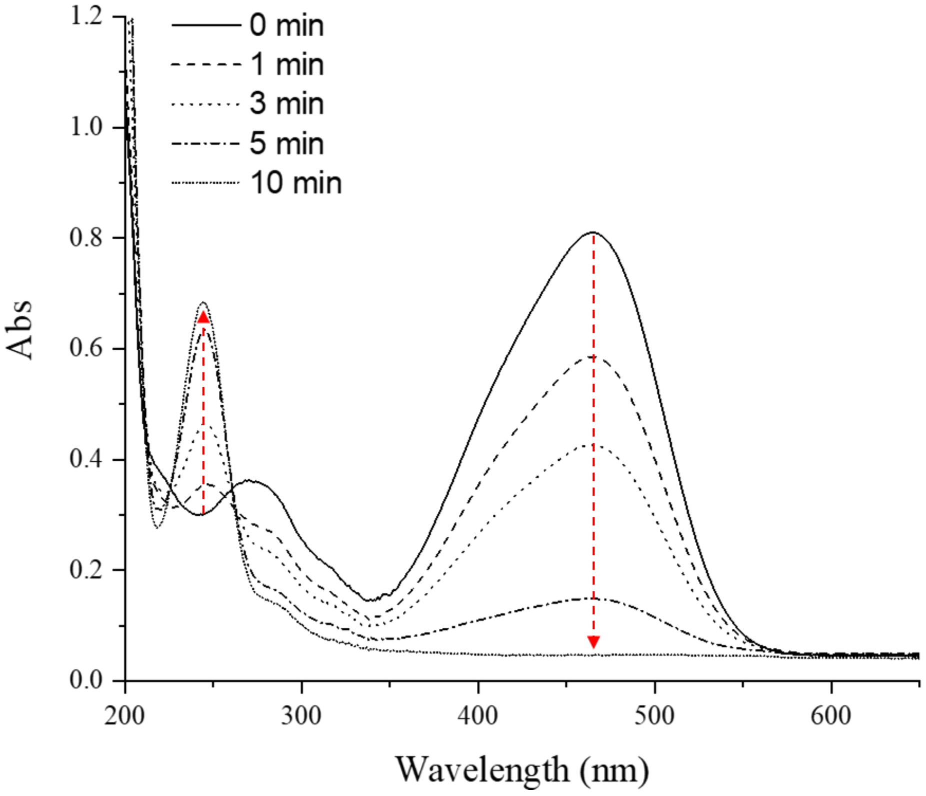

0 min
1 min
3 min
5 min
10 min
Abs
Wavelength (nm)
1.2
1.0
0.8
0.6
0.4
0.2
0.0
200
300
400
500
600

Figure S5.

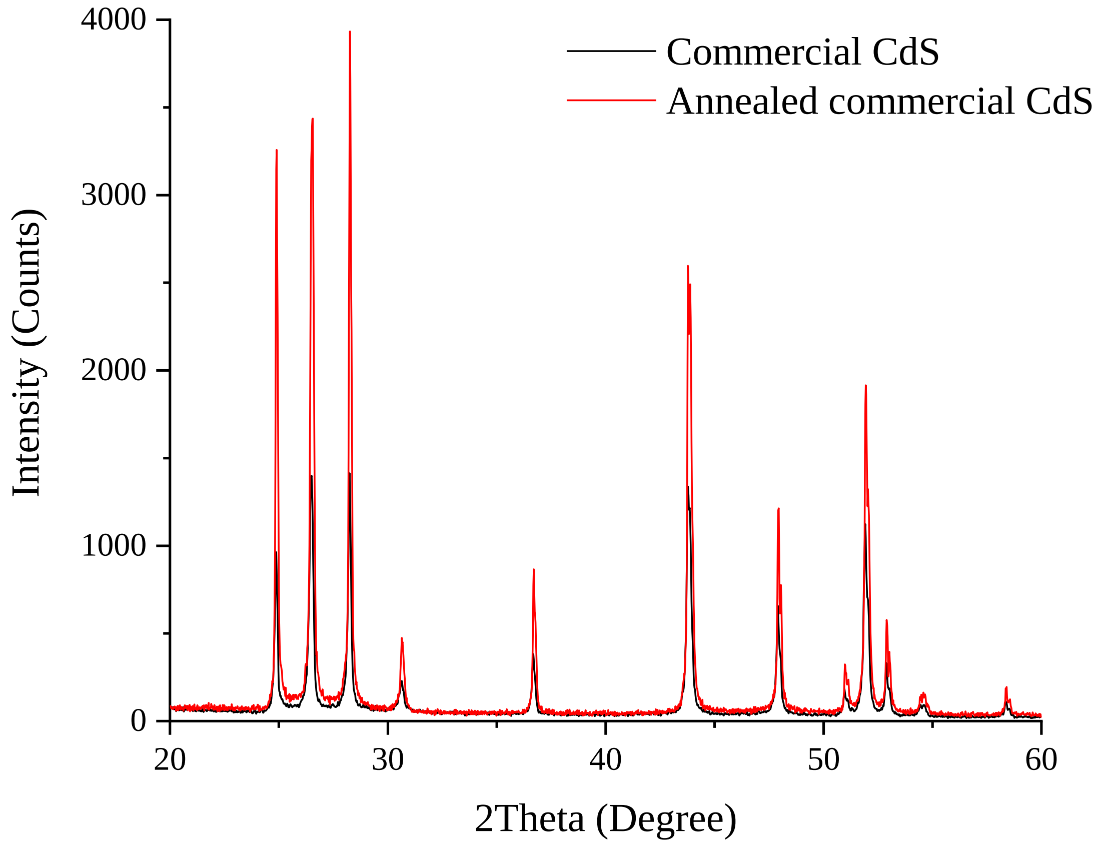

4000
3000
2000
1000
0
Intensity (Counts)
20
30
40
50
60
2Theta (Degree)
Commercial CdS
Annealed commercial CdS

Figure S6

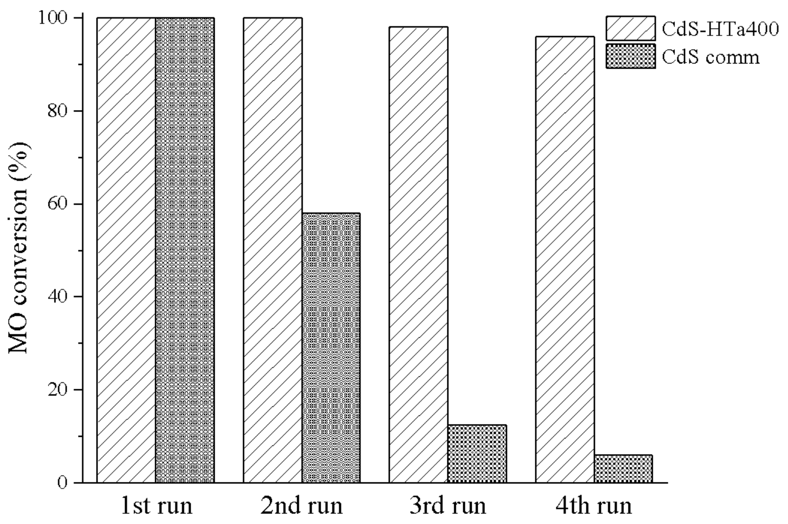
100
80
60
40
20
0
MO conversion (%)
1st run
2nd run
3rd run
4th run
CdS-HTa400
CdS comm

Figure S7.

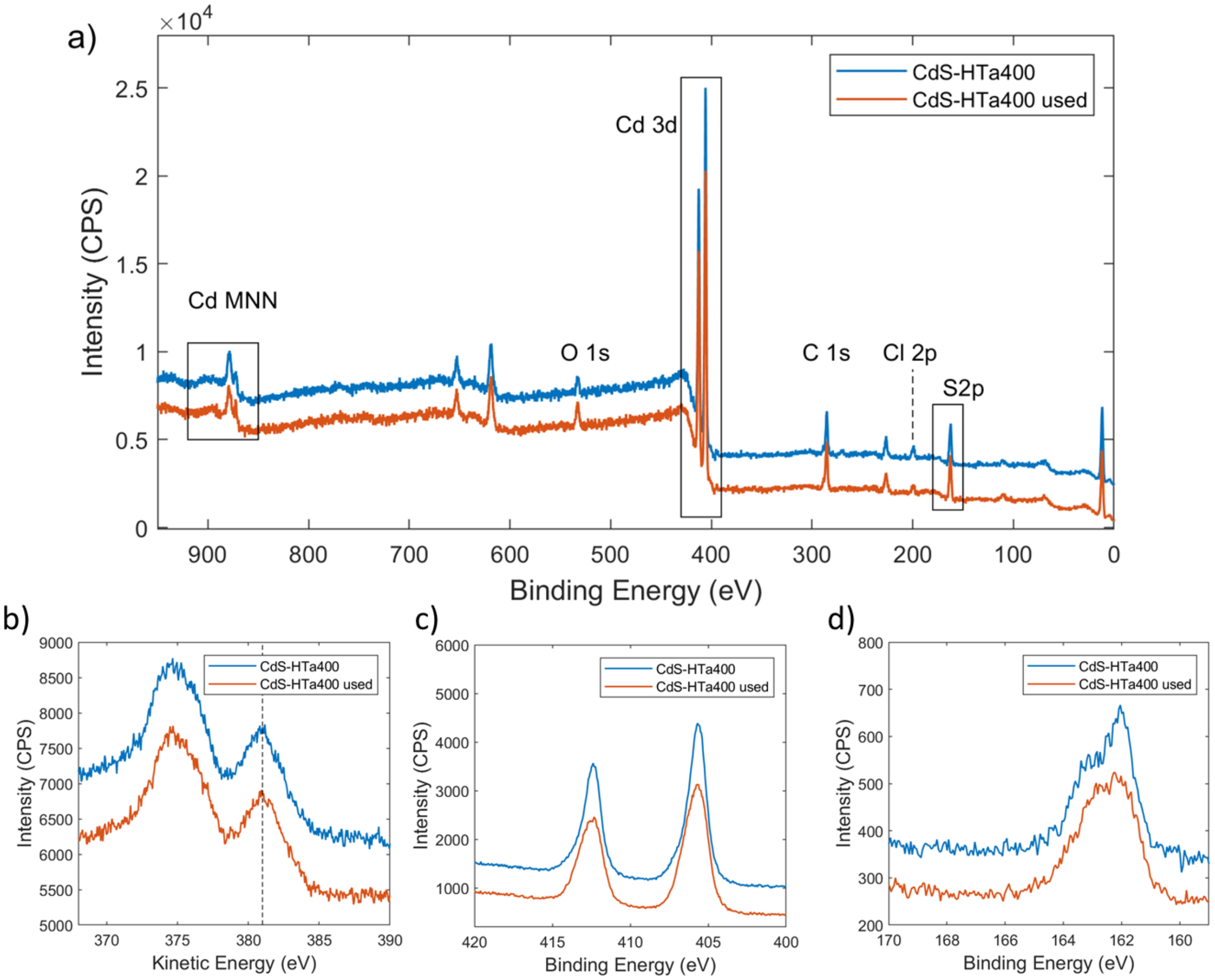

Figure S8.

Table S2.

Elements	C 1s 284.4 eV	O 1s 532.5 eV	Cd 3d 5/2 405.6 eV	S 2p 162.1 eV	Cl 2p 199 eV	Cd/S
CdS-HTa400	40.8 ± 1.0	7.4 ± 0.7	24.9 ± 0.8	21.2 ± 0.8	5.6 ± 0.5	1.2 ± 0.1
CdS-HTa400 used	46.2 ± 1.0	10.2 ± 0.7	21.1 ± 0.8	19.4 ± 0.8	3.1 ± 0.5	1.1 ± 0.1
